# Auxin Controlled by Ethylene Steers Root Development

**DOI:** 10.3390/ijms19113656

**Published:** 2018-11-20

**Authors:** Hua Qin, Rongfeng Huang

**Affiliations:** 1 Biotechnology Research Institute, Chinese Academy of Agricultural Sciences, Beijing 100081, China; qinhua@caas.cn; 2 National Key Facility of Crop Gene Resources and Genetic Improvement, Beijing 100081, China

**Keywords:** auxin, ethylene, phytohormones, root development, crosstalk

## Abstract

Roots are important plant ground organs, which absorb water and nutrients to control plant growth and development. Phytohormones have been known to play a crucial role in the regulation of root growth, such as auxin and ethylene, which are central regulators of this process. Recent findings have revealed that root development and elongation regulated by ethylene are auxin dependent through alterations of auxin biosynthesis, transport and signaling. In this review, we focus on the recent advances in the study of auxin and auxin–ethylene crosstalk in plant root development, demonstrating that auxin and ethylene act synergistically to control primary root and root hair growth, but function antagonistically in lateral root formation. Moreover, ethylene modulates auxin biosynthesis, transport and signaling to fine-tune root growth and development. Thus, this review steps up the understanding of the regulation of auxin and ethylene in root growth.

## 1. Introduction

Roots are important plant ground organs, which are crucial for plant survival and performs a wide range of functions, such as absorbing water and nutrients, supporting the plant body and interactions with soil microbiota. Generally, the root system consists of two principal root types: the primary root (PR), which is initiated during embryo development [1] and secondary roots, which form post-embryonically. These secondary roots encompass both lateral roots (LR), which develop as branches of the primary root and adventitious roots (AR), which develop on non-root tissue such as the hypocotyl, stems and leaves [2]. The root architecture of monocots and dicots is highly distinct. The dicotyledonous plant has a tap-root system with a central primary root and lateral roots, whereas monocotyledonous plants, such as rice (*Oryza sativa*) and maize (*Zea mays*), have fibrous root systems composed of a primary root, lateral roots and crown roots (also known as adventitious roots) [3].

In higher plants, root growth is maintained by coordinating cell proliferation and differentiation [4,5]. Phytohormones have been known to play a crucial role in the regulation of root growth [6,7,8,9]. Recent studies in *Arabidopsis* root have shown that different hormones control organ growth by regulating specific growth processes such as cell proliferation, differentiation or expansion in distinct tissues [9,10,11]. Plant hormones such as auxin and ethylene have been shown to be involved in root growth through a range of complex interactions [8].

Indole-3-acetic acid (IAA), the main auxin in plants, regulates almost every aspect of plant growth and development [12]. Genetic and biochemical studies indicated that tryptophan (Trp) is the main precursor for IAA in plants [13]. There are three pathways for IAA biosynthesis from Trp in plants: the indole-3-pyruvic acid (IPyA) pathway, the indole-3-acetamide pathway and the indole-3-acetaldoxime pathway [14]. The IPyA pathway has been proposed as the most important pathway to produce auxin in plant [15,16,17]. Once auxin is produced, it is perceived by TRANSPORT INHIBITOR RESPONSE 1 (TIR1) and AUXIN SIGNALING F-BOX PROTEIN (AFB). IAA directly interacts with the F-box protein TIR1 and promotes the degradation of the Aux/IAA transcriptional repressors to activate diverse auxin responsive genes [18,19]. Recent studies have shown that auxin biosynthesis, transport and auxin-dependent signaling processes all affect root development [13,20].

Ethylene is a simple and very important gaseous phytohormone that modulates multiple plant growth and development processes [21]. Ethylene is synthesized from methionine via a simple linear pathway, in which 1-aminocyclopropane-1-carboxylic acid (ACC) synthase (ACS) and ACC oxidases (ACO) function as key enzymes [22]. Ethylene is sensed by a family of endoplasmic reticulum-located receptors, which are negative regulators of the signaling pathway [23,24]. In the absence of ethylene, the active receptors recruit a Raf-like protein kinase, CONSTITUTIVE TRIPLE RESPONSE 1 (CTR1), to phosphorylate the C-terminal domain of ETHYLENE INSENSITIVE 2 (EIN2), thus causing the proteasome degradation of EIN2 by F-box proteins and repression of the downstream ethylene response [25,26,27]. In the presence of ethylene, ethylene binding to the receptors inhibits the interaction with CTR1, resulting in that CTR1 cannot phosphorylate EIN2. Unphosphorylated EIN2 is cleaved by unknown proteases and the EIN2 C-terminus translocate into the nucleus [25,28,29] or into the P-body [30,31]. In the P-body, the EIN2 C-terminus mediates translational repression of EIN3-BINDING F-BOX 1 (EBF1) and EBF2 [30,31,32]. In the nucleus, the EIN2 C-terminus transduces signals to the transcription factors EIN3 and EIN3-LIKE1 (EIL1), which are sufficient and necessary for activation of many ethylene-response genes. These changes ultimately cause different physiological responses [33,34,35]. Due to the pivotal regulation of ethylene and auxin in root growth, in which the ethylene elevates auxin accumulation and to trigger TIR1/AFB2-mediated signal transduction [36,37,38,39], here in this review we will focus our interest on the recent advances of ethylene, auxin and their crosstalk during root development, providing a different angle for analyzing the mechanisms of plant root development.

## 2. The Coordination of Ethylene and Auxin in Primary Root Growth

The primary root, initiated during embryo development, develops shortly after germination and is a fundamental part of the root system that absorbs mineral nutrients and provides mechanical support for shoot growth [1]. Plant root is characterized by a series of developmental zones: the meristematic zone (MZ), transition zone (TZ), elongation zone (EZ) and growth terminating zone (GTZ) [40,41]. In the center of the root tip there is a quiescent center (QC), which is mitotically inactive and functions as organizer of the root stem cell niche. [42].

Auxin is known to exert an inhibitory role on primary root growth. An auxin gradient, established by local auxin biosynthesis and transport, is important for primary root growth. Auxin synthesized in roots via the IPyA pathway is crucial in normal root elongation and root gravitropic responses [43]. Disruption of YUC genes and/or TAA genes can cause moderate to very severe root defects in *Arabidopsis*. For example, the *taa1 tar1 tar2* triple mutant in *Arabidopsis* fails to make root meristem during embryogenesis [44]; similar phenotypes were observed in *yuc1 yuc4 yuc10 yuc11* quadruple mutant as well [45]. But *yuc3 yuc5 yuc7 yuc8 yuc9* quintuple mutant (*yucQ*) displays very short primary roots and agravitropic root growth. Histological analysis showed that *yucQ* had much smaller meristems, with enlarged cells [43]. In addition, overexpressing any member of the YUC family in *Arabidopsis* leads to primary root growth inhibition [15,43,46], indicating that basal levels of endogenous auxin are required to maintain normal root growth. In rice, disruption of the *FISH BONE* gene, an orthologue of *TAA1*, displays pleotropic phenotypes including agravitropic roots, long primary roots, few crown roots and a lack of lateral roots [47]. Overexpression of *OsYUC* genes enhances the development of crown roots, shortened primary roots and over proliferation of root hairs [48,49]. Knock-down *OsYUC1* expression results in the inhibition of root formation and elongation [48]. In woodland strawberry (*Fragaria vesca* L.), silencing of *YUCCA6* affected various post-embryonic organ developmental steps including root formation [50]. All these results evidence that auxin synthesized by the IPyA pathway plays important roles in primary root development.

Inhibition of root growth is also one of the characteristic effects of ethylene or its precursor ACC. In *Arabidopsis*, exogenous application of ethylene or ACC inhibits the primary root elongation [51]. Mutants of *ETHYLENE OVERPRODUCER* (*eto1*) and *ctr1*, with an enhanced ethylene biosynthesis or signaling, respectively, exhibit short primary roots [52,53,54]. In contrast, the inhibitory effect of ethylene on primary root growth is abolished in ethylene insensitivity mutants, such as *ETHYLENE RESISTANT 1* (*etr1*), *ein2*, *ein3 eil1* or in the presence of ethylene inhibitors, such as the biosynthesis inhibitor AVG [51] and pyrazinamide (PZA) [55], the perception inhibitor silver nitrate (AgNO_3_) [51] and 1-methylcyclopropene (1-MCP) [56]. In rice, ethylene inhibits primary root elongation [39,57,58]. Loss-of-function mutation of rice ethylene receptors shows significant shorter primary roots and a moderately enhanced ethylene response [6,59,60]. By screening rice ethylene response mutants, MHZ7/OsEIN2 and MHZ6/OsEIL1 have been identified [57,61]. Mutation of *OsEIN2/OsEIL1* leads to completely insensitive to ethylene in primary root growth, *OsEIN2/OsEIL1*-overexpression rice seedlings showed very short and extremely twisted primary roots [57,61]. MHZ3, positively regulates the ethylene signaling by interacting with the OsEIN2 Nramp-like domain and regulating the stability of the OsEIN2 protein [62]. Loss-of-function mutation of *MHZ3* leads to ethylene insensitivity in etiolated rice seedlings, whereas *MHZ3* overexpression lines exhibited slightly but significantly longer coleoptiles and shorter primary roots when grown in the air [62]. In other monocotyledonous plants, such as maize, wheat, sorghum and *B. distachyon*, ethylene treatment also inhibits the primary root growth [58].

Ethylene affects root growth at two different levels. First, ethylene inhibits cell proliferation in the root apical meristem [11]. Second, ethylene inhibits cell expansion in the root elongation zone [51,63]. The reports of ethylene effects on cell division in roots are conflicting. Ruzicka et al. [51] showed that ethylene affects root growth primarily by regulating the elongation of cells that leave the root meristem but without impact on the root meristem size. Recent studies showed that ethylene negatively regulates root meristem size through inhibiting cell proliferation in the root meristem [11]. Through a targeted expression approach to map the tissue sites of ethylene growth regulation found that the epidermis is the main site of ethylene action controlling plant growth in both roots and shoots [10]. Taken together, ethylene inhibits primary root growth by inhibiting cell proliferation in meristem zone and cell elongation in elongation zone.

The auxin–ethylene crosstalk during root growth has been proposed based on several studies [36,37]. In *Arabidopsis*, ethylene application promotes the expression of IAA biosynthetic genes and IAA levels [44,51,64]. In ethylene-treated seedlings, an overall increase of auxin response at the root tip was observed and this is also reflected in direct auxin measurements [36,51]. By screening for mutants that display ethylene defects only in roots, some *WEAK ETHYLENE INSENSITIVE* (*WEI*) genes were identified, such as *WEI2/ASA1*, *WEI7/ASB1* and *WEI8/TAA1* [44,64]. *WEI2/ASA1* and *WEI7/ASB1* encode subunits of anthranilate synthase, a rate-limiting enzyme in Trp biosynthesis. Upregulation of *WEI2/ASA1* and *WEI7/ASB1* by ethylene results in the accumulation of auxin in the tip of primary root, whereas loss-of-function mutations in these genes prevent the ethylene-induced auxin increase [64]. *WEI8/TAA1* encodes a long-anticipated tryptophan aminotransferase, a key enzyme in the IPyA pathway, catalyzes the conversion Trp to IPA. Analysis of *WEI8/TAA1* and its paralogues revealed a link between local auxin production and tissue-specific ethylene effects [44]. The same phenotypes were also observed in simultaneously inactivated YUC mutant roots, such as *yucQ*. YUC catalyzes the conversion IPA to IAA, a rate-limiting step in the IPyA pathway [17]. These mutants underscore the link between ethylene signaling and auxin biosynthesis. However, it is not clear how ethylene signal is transmitted to auxin. Recent studies have shown that several transcription factors in the ethylene signaling pathway, such as EIN3, ETHYLENE RESPONSE FACTOR 1 (ERF1) and PHYTOCHROME INTERACTING FACTOR 4 (PIF4), function as crosstalk nodes between ethylene and auxin in root growth [65,66,67,68]. Particularly, PIF4 affects auxin-mediated growth by directly controlling the expression of *TAA1* and *YUC* genes [65,66]. EIN3 directly binding to the *YUC9* promoter to regulate auxin accumulation in root transition zone and root growth inhibition [67]. ERF1 binds the promoter of *ASA1* in order to regulate auxin biosynthesis and ethylene-induced root growth inhibition [68]. Another interaction between ethylene and auxin biosynthesis was discovered in a chemical genetic strategy, using l-kynurenine, a small molecule that represses nuclear accumulation of the EIN3 transcription factor and *TAA/TAR* was identified as a target for l-kyn, to decrease ethylene-induced auxin biosynthesis and ethylene responses in roots [69]. Using yucasin [5-(4-chlorophenyl)-4*H*-1,2,4-triazole-3-thiol], an inhibitor of YUC activity, suppresses ethylene-inhibited root growth [39,70,71]. Recent studies in rice showed that exogenous application of yucasin largely recovered the short and coiled primary root phenotype of *OsEIN2* and *OsEIL1* overexpression transgenic lines and OsEIL1 directly activates the expression of *OsYUC8* to modulate auxin biosynthesis and ethylene-inhibited primary root elongation [39].

In addition to auxin biosynthesis mutants, mutants related to auxin transport, perception and signaling also display abnormal responses to ethylene [37,51,64,72]. In *Arabidopsis*, mutants of auxin receptor *tir1* and auxin response *AUXIN RESISTANT axr2-1* and *axr3-1*, mutants in *IAA7* and *IAA17*, respectively, were shown an ethylene-resistance root phenotype [37,51]. In rice, soil-surface rooting 1 (SOR1), which is a RING finger E3 ubiquitin ligase, controls root-specific ethylene responses by modulating Aux/IAA protein stability [38]. In conclusion, ethylene-regulated primary root growth requires auxin biosynthesis, perception, transport, or signaling.

## 3. The Integration of Ethylene and Auxin in Lateral Root Growth

Lateral roots are an essential component of root system architecture that maximizes the ability of the root system to acquire nutrients, therefore enabling the plant to adapt readily to the changes of the environment cues [73,74]. Lateral root development is initiated from asymmetric divisions of the pericycle founder cell of primary root and the whole process of development has been well established [75,76].

It is well known that the phytohormone auxin is involved in every stage of lateral root formation [75,77]. Endogenous auxin biosynthesis, polar auxin transport and auxin-dependent signaling processes all affect lateral root formation [78,79,80,81]. In *Arabidopsis* roots, gain-of-function *yuc* mutant (*yuc1D*), *superroot1* (*sur1*) and *sur2* showed an increase in endogenous auxin levels and lateral root number [46,82]. The mutants with defects in auxin transport, such as *auxin resistant 1* (*aux1*), *like aux1 3* (*lax3*), *pin-formed* (*pin*) multiple mutants, *gnom* and *auxin resistant 4* (*axr4*), have reduced lateral root number or disturbed lateral root primodium [82]. The rice *osaux1* mutant has a reduced number of lateral roots, accompanied by decreased endogenous auxin levels [79,83,84]. Furthermore, an elevated auxin level causes degradation of Aux/IAAs transcriptional regulators and releases ARFs [18,19], which promote lateral root proliferation [82,85]. A series of *Arabidopsis* gain-of-function Aux/IAA mutants, such as *iaa1*, *iaa3*, *iaa14/solitaryroot 1* (*slr1*), *iaa18*, *iaa19*/*massugu 2* (*msg2*), *iaa28* [82,86] and *osiaa11*, *osiaa13* and *osiaa23* from rice, showed aberrant lateral roots, reduced lateral root number, or even complete absence of lateral root formation [87,88,89]. Accordingly, ARF mutants, such as *arf7 arf19* double mutant, showed complete absence of lateral root formation [82].

Ethylene also plays an important role during the initiation and growth of lateral roots. For reviews of ethylene effects on lateral roots development, we refer to [90]. Treatment with ethylene or ACC reduces lateral root initiation in both *Arabidopsis* and tomato [91,92]. Similarly, enhanced ethylene synthesis (in *eto1* mutant) or signaling (in *ctr1* mutant) reduced lateral root formation [91]. By contrast, ethylene-insensitive mutants (*etr1* and *ein2*), form an increased number of lateral roots [91], suggesting that ethylene exerts an inhibitory effect on lateral root formation.

Auxin and ethylene have antagonistic effects in lateral root development. Ethylene reduces DR5erv:GFP expression in the regions where lateral roots emerge, suggesting a locally reduced auxin responsiveness [93]. Mutants in auxin influx and efflux proteins, such as *aux1*, *lax3*, *pin3* and *pin7*, were less sensitive to the inhibition of lateral root formation and stimulation of auxin transport following treatment with ACC [91,93]. By contrast, *pin2* and *abcb19* mutants exhibited normal responses to ACC [93]. *Arabidopsis* seedlings show a local depletion of PIN3 and PIN7 abundance in the region just below developing lateral root primordia, resulting in a local auxin accumulation at the lateral root primordia, thus promoting the formation of lateral roots. ACC treatment increased the abundance of transcripts *PIN3* and *PIN7*, thus increasing rootward auxin transport, which suppresses the local auxin maxima at lateral root primordia, inhibiting their outgrowth [93,94].

## 4. The Role of Ethylene and Auxin in Adventitious Root Growth

Adventitious root, also known as crown root, constituting the major part of the monocotyledonous root system, emerges from any non-root tissue and is produced both during normal development and in response to stress conditions, such as flooding, nutrient deprivation and wounding during the growing period [95].

Similar to other development processes, adventitious root formation is highly dependent on auxin action [2]. Overexpression *YUCCA* genes in rice cause massive proliferation of crown roots. On the other hand, disruption of *TAA1* greatly reduces crown root development [47,49]. Many genes essential for crown root development in rice are involved in the auxin-signaling pathway. For example, *CROWNLESS ROOT 1* (*CRL1*)*/ADVENTITIOUS ROOT LESS 1* (*ARL1*) encodes an ASYMMETRIC LEAVES 2 (AS2)/LATERAL ORGAN BOUNDARIES (LOB) transcription factor, which is transcriptionally regulated by the AUXIN RESPONSE FACTOR 16 (ARF16), acts as a positive regulator for crown root formation [96,97]. CRL4/OsGNOM1 plays an important role in crown root emergence by its influence on the polar localization of the auxin efflux carrier PIN1 [98]. The *crl5* mutant produced fewer crown roots and displayed impaired initiation of crown root primordia. Its expression can also be induced by exogenous auxin treatment and may be a direct target of an ARF [99]. CRL6 influences crown root formation by regulating primordial initiation and development. It was shown that the expression of *OsIAA* genes were down-regulated in *crl6*, linking CRL6 to auxin regulatory network [100]. The *WUSCHEL-related Homeobox* (*WOX*) gene, *WOX11*, is involved in the activation of crown root emergence and growth. Its expression is induced by auxin and functions downstream IPyA pathway [3,49,101,102,103]. These studies show that the auxin is essential for crown root development in rice.

Ethylene is another important phytohormone in root development through interacting with auxin. However, ethylene function in crown root development remains unknown. In rice, OsERF3, which encodes an AP2/ETHYLENE-RESPONSIVE FACTOR (ERF) protein, negatively regulates ethylene biosynthesis and act as a WOX11-interacting partner involved in rice crown root development [3,104]. Moreover, the expression of OsERF3 is induced by auxin and ACC treatment, suggesting a potential relationship between ethylene and auxin in crown root development. Further research should focus on the ethylene effect on crown root development and illuminating the underlying molecular mechanism.

## 5. The Contribution of Ethylene and Auxin in Root Hair Growth

Plant root hairs are unicellular extensions of root epidermal cells that increase the root surface to enhance water and nutrient uptake and improve soil anchorage. Multiple phytohormones, including auxin and ethylene, play vital roles in regulating cell development in the root epidermis [105,106]. Constitutive activation of ethylene signaling or exogenous application of ethylene or ACC promotes root hair elongation. By contrast, the root hairs of ethylene-insensitive mutants and seedlings treated with inhibitor of ethylene synthesis or signaling are significantly shorter [105,107,108]. Root hair elongation is enhanced by exogenous auxin treatment [51]. Similarly, enhanced auxin synthesis (in *sur1* and *yuc1D* mutants) promoted root hair elongation [13,46]. Moreover, ethylene-signaling mutants and auxin-signaling mutants develop fewer and shorter root hairs [8]. These data strongly indicate that auxin and ethylene are both required for root hair elongation. Further studies found that auxin is able to rescue root hair defects in the *ein2-1* mutant and mutant *aux1* on an *eto1* background suppressed the long-root-hair phenotype of *eto1* mutant [109]. Recent studies demonstrated that several ARFs, which are central transcriptional regulators of auxin signaling, bind the *ROOT HAIR DEFECTIVE 6-LIKE 4* (*RSL4*) promoter and directly activate its expression [110], thus providing the molecular link between auxin signaling and transcriptional control of root hair development [105]. RSL4 also involved in ethylene-promoted root hair growth, ethylene-activated transcription factor EIN3 physically interacts with ROOT HAIR DEFECTIVE 6 (RHD6), a well-documented positive regulator of hair cells and that the two factors directly co-activate *RSL4* to promote root hair elongation [107]. However, whether ARFs and EIN3 act synergistically or competitively to activate RSL4 in root hair development is unclear.

## 6. Conclusions and Future Perspectives

**Figure 1:**
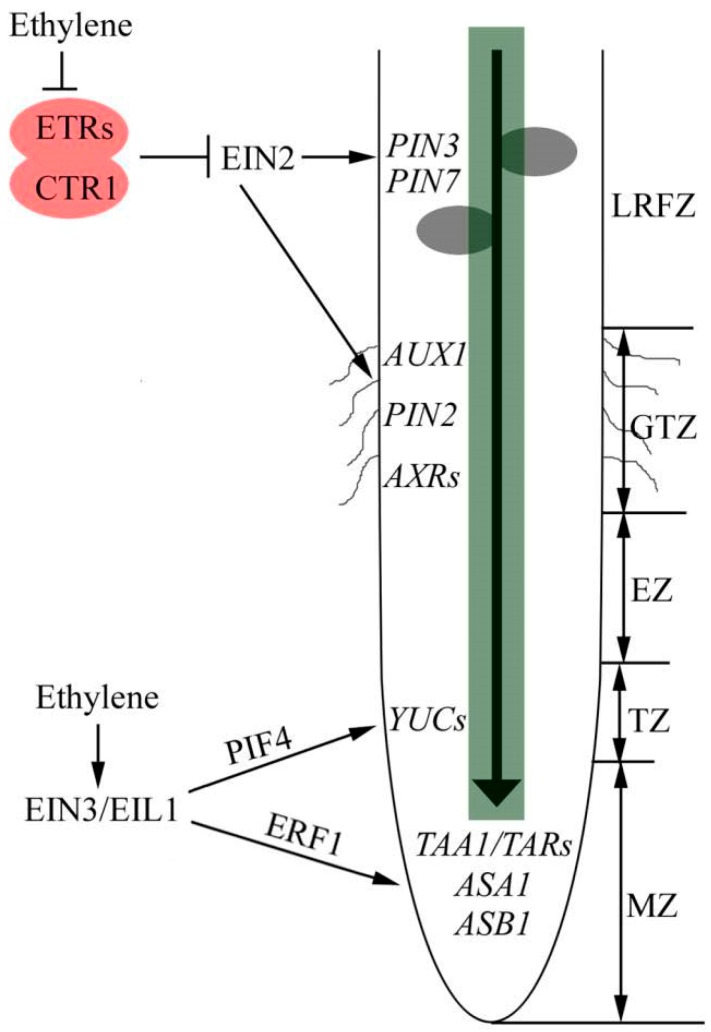
Ethylene directs auxin to control root growth. The growth zones in root apex, including meristematic zone (MZ), transition zone (TZ), elongation zone (EZ) and growth terminating zone (GTZ). Ethylene promotes auxin biosynthesis in MZ and TZ through *ASA1*, *ASB1*, *TAA1/TARs* and *YUCs*, leading to inhibition of primary root elongation. ERF1 and PIF4 function as crosstalk nodes between ethylene and auxin in this process. In GTZ, ethylene promotes root hair initiation through modulation of the auxin levels mediated by auxin transporters. In the lateral root-forming zone (LRFZ), ethylene increases rootward auxin transport by increasing transcription and translation of *PIN3* and *PIN7* in the central cylinder, which prevent the localized accumulation of auxin needed to drive lateral root formation. Arrows indicates positive regulation, T sharp symbol indicates negative regulation, and waves in GTZ zone represent root hairs.

Over the years, the crosstalk between ethylene and auxin in *Arabidopsis* root development has been elucidated. However, their interaction in other plants is still unclear. In this review, we summarize the recent advances in the study of auxin–ethylene crosstalk in plant root development (Figure 1), step up our understanding of the mechanisms of plant root development.

Plant root systems adapt rapidly to environmental changes and enable the plant to survive adverse conditions. In response to environmental cues, root growth is adapted through the modulation of endogenous auxin levels [111]. However, the underlying mechanisms of plants perceiving environmental changes and translating the cues into adaptive responses involved in the integrative regulation of auxin and ethylene in this process is largely unclear. Moreover, root growth is maintained by coordinating cell proliferation and differentiation [4,5]. Recent studies showed that epidermis is a key cell type required for ethylene-mediated growth inhibition [10]. Whether ethylene is required in multiple cell types for crosstalk with auxin or whether its effect is direct or indirect remains unknown. Cell type-specific promoters allow scientists to target the expression of a desired effector protein to particular cell types and reveal the necessity of the ethylene and auxin in a given cell type.

Ethylene and auxin are known to interact in the regulation of root growth. Several factors function as crosstalk nodes between ethylene and auxin have been identified in *Arabidopsis*, such as EIN3, ERF1 and PIF4 [65,66,67,68]. However, information in crops is limited. Moreover, hormonal crosstalk is not linear and should be tackled in a multidimensional space, sparking that the spatial and temporal relationships between the two hormones and the interdependent relationships with other hormones should be focused. The use of new chemicals known to target specific hormonal pathway components and the advent of high-throughput nucleotide sequencing techniques and CRISPR-CAS technology allowing generating specific mutants will easy to elucidate the underlying mechanisms of plant root development.
